# Reduced Interhemispheric Coherence after Cerebellar Vermis Output Perturbation

**DOI:** 10.3390/brainsci10090621

**Published:** 2020-09-08

**Authors:** Elena Laura Georgescu Margarint, Ioana Antoaneta Georgescu, Carmen-Denise-Mihaela Zahiu, Alexandru Răzvan Șteopoaie, Stefan-Alexandru Tirlea, Daniela Popa, Ana-Maria Zagrean, Leon Zagrean

**Affiliations:** 1 Division of Physiology and Neuroscience, Carol Davila University of Medicine and Pharmacy, 050474 Bucharest, Romania; laura.georgescu@umfcd.ro (E.L.G.M.); antoaneta.georgescu@drd.umfcd.ro (I.A.G.); razvan.steopoaie@stud.umfcd.ro (A.R.Ș.); stefan.tirlea@stud.umfcd.ro (S.-A.T.); dpopa@bio.ens.psl.eu (D.P.); leon.zagrean@umfcd.ro (L.Z.); 2 Institut de biologie de l’Ecole normale supérieure (IBENS), Ecole normale supérieure, CNRS, INSERM, PSL Research University, 75005 Paris, France

**Keywords:** cerebellum, interhemispheric coherence, kainate, mice, oscillation, motor cortex, compensation, dystonia

## Abstract

Motor coordination and motor learning are well-known roles of the cerebellum. Recent evidence also supports the contribution of the cerebellum to the oscillatory activity of brain networks involved in a wide range of disorders. Kainate, a potent analog of the excitatory neurotransmitter glutamate, can be used to induce dystonia, a neurological movement disorder syndrome consisting of sustained or repetitive involuntary muscle contractions, when applied on the surface of the cerebellum. This research aims to study the interhemispheric cortical communication between the primary motor cortices after repeated kainate application on cerebellar vermis for five consecutive days, in mice. We recorded left and right primary motor cortices electrocorticograms and neck muscle electromyograms, and quantified the motor behavior abnormalities. The results indicated a reduced coherence between left and right motor cortices in low-frequency bands. In addition, we observed a phenomenon of long-lasting adaptation with a modification of the baseline interhemispheric coherence. Our research provides evidence that the cerebellum can control the flow of information along the cerebello-thalamo-cortical neural pathways and can influence interhemispheric communication. This phenomenon could function as a compensatory mechanism for impaired regional networks.

## 1. Introduction

Interhemispheric communication in the brain is fundamental for the generation of synchronized neural activities, which favor task-related high performance and efficient cognitive and emotional abilities. The entire brain activity is an organized system of rhythms responsible for different physiological functions [1]. Human studies have shown hemispheric asymmetry in motor control when analyzing the primary motor cortex and supplementary motor area. In these studies, wrist movements generated lower coherence levels between the two cerebral cortical structures, suggesting a lack of symmetry in their coupling. This phenomenon was also found in other motor and psychiatric diseases such as Parkinson’s disease and attention-deficit hyperactive disorder (ADHD) [2,3,4,5].

The cerebellum is involved in the functional regulation of the neocortex’s motor areas by influencing the brain oscillations through the cerebello-thalamo-cortical circuit [6,7,8]. Recently, more and more evidence has emerged that demonstrates the cerebellum’s ability to operate in a much more sophisticated manner than initially thought. The cerebellum has been found to generate efficient and harmonized activities, using local networks that synchronize via particular membrane and synaptic mechanisms [9]. There are multiple gap junctions at the inferior olive level and between the stellate cells from the molecular layer [10], but also between the Golgi cells from the granular layer [11]. This particular organization indicates that the cerebellum holds the necessary functional structure for high neuronal synchronization and can even generate rhythmic activities using the local microcircuits [12,13]. The neurons found in the olivo-cerebellar system can thus exhibit an autorhythmic firing pattern and create oscillations, especially in the theta band. This firing pattern is also influenced by cerebellar Golgi cells [14].

The cerebellum has been found to also have a role in dystonia, a heterogeneous motor disorder characterized by sustained muscle contractions and sometimes abnormal repetitive movements or postures [15,16]. Several animal and human studies showed that changes in the activity, structure, connectivity with other brain regions and plasticity of the cerebellum are linked with dystonia [17,18,19,20,21,22]. A recent study demonstrated that a protocol of continuous theta-burst stimulation could not decrease the excitability of the motor cortex in subjects with cervical dystonia, demonstrating altered cerebellar plasticity and altered connectivity [23]. Dystonia can be triggered pharmacologically by applying kainate on the surface of the cerebellum, which alters cerebellum signaling. Kainate is a potent excitatory cyclic analog of L-glutamate and agonist of kainate ionotropic glutamate receptors [24,25,26]. Several studies have successfully mapped the localization of kainate receptors. They can be found at different levels of expression in the amygdala [27], basal ganglia [28], and cerebellum [29].

In this study, we evaluated the cerebellum involvement in motor cortex interhemispheric communication in the case of dystonia induced in mice by repeatedly activating the vermis’s surface with kainate for five consecutive days. Moreover, we searched for possible correlations between dystonic behavior and interhemispheric coherence.

## 2. Materials and Methods

### 2.1. Animals

The study was conducted after the consent of the ethical committee of Carol Davila University of Medicine and Pharmacy, Bucharest, Romania (ethical committee code: PO-35-F-03), respecting The European Communities Council Directive 2010/63/EU of the European Parliament concerning the ethical conduct for animal experiments. Tests were carried out on 12–16-week-old Swiss albino male mice (*n* = 10), weighing 35–47 g, that were kept in standard conditions with free access to water and food.

### 2.2. Surgery

**Figure 1:**
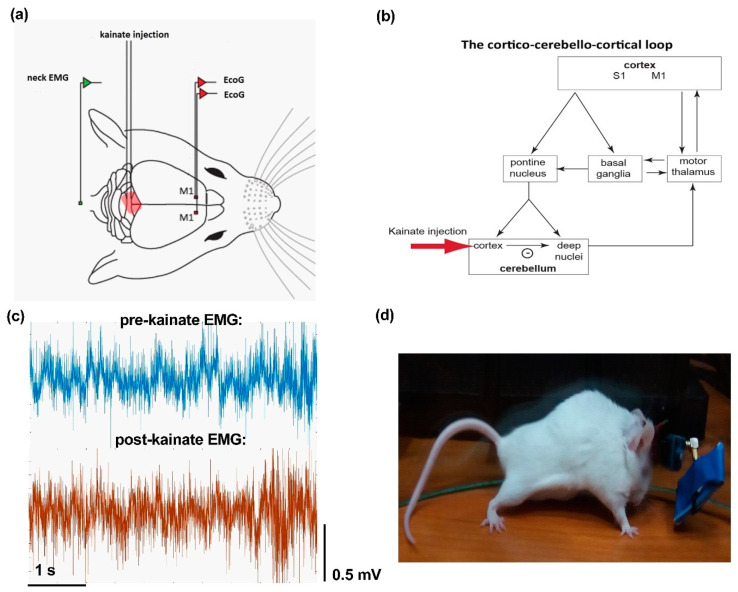
(**a**) Schematic representation of the experiment setup (adaptation after Georgescu et al. [15]); placement of the electrodes for the left and right primary motor cortices (M1) electrocorticograms (ECoGs). (**b**) Diagram of the cortico-cerebellar-cortico-circuit (S1 is an acronym for sensorimotor cortex) and indication of the site of kainate application. (**c**) Example of electromyogram (EMG) traces before and after the kainate cerebellar vermis administration. (**d**) The phenotypic effect of the kainate administration with intense truncal muscle contractions.

Mice were anesthetized with 3–4% isoflurane (RomPharm, Romania) for induction and 2% isoflurane for the rest of the surgery. Buprenorphine (Sigma-Aldrich, Saint Louis, MS, USA, 50 µg/kg body weight) was administrated before and every 24 h after surgery for 3 days to control the pain. The level of anesthesia was adjusted using the withdrawal reflex threshold to a pain stimulus. The body temperature was maintained at 37 °C with a surgical heating pad (FHC Inc., Maine ME, USA). A volume of 1 ml of 1% lidocaine was injected subcutaneously on the midline of the scalp, then the soft tissue was removed, and the skull was exposed. For implanting the electrodes for electrocorticogram (ECoG) recordings, three craniotomies (1 mm diameter) were performed with an electric drill under stereotaxic guidance (David Kopf Instruments, California CA, USA) as follows: two at the level of the left and right primary motor cortices (2.2 mm anterior to the bregma and 2.2 mm lateral, left and right, to the sagittal suture), and one for the ground, reference electrode (2 mm posterior and 2 mm to the right of lambda). The electrodes were manufactured from nichrome bendable wire of 0.15 mm width (Kanthal, Hallstahamar, Sweden), and implanted after de-insulating their contact regions with the tissues and with the pins of the W2100-HS4-opto Headstage (Multi-Channel Systems) to increase the signal’s quality. The electromyogram (EMG) electrode was inserted in the neck muscles (Figure 1a). The wires were attached to the pins and then fixed with Super Bond glue (Dental Adhesive Resin Cement, Sun Medical CO, Japan) and with dental adhesive (Pi-Ku-Plast HP 36, Bredent GmbH, Germany). A guide cannula used for kainate administration was fixed vertically on the cerebellar lobule 6 on the vermis (Figure 1a,b), under stereotaxic control.

### 2.3. Kainate Microinjection and ECoG and EMG Recordings

Mice were left for 4 days to recover post-surgery before the beginning of the experiments. ECoG and EMG recordings were conducted in awake animals in an open field using a Multi-Channel Systems W2100 wireless interface board (Figure 1a). The acquisition was performed with a 4-channel W2100-HS4-opto Headstage (the total weight of the headstage was 1.9 g + 3.8 g for the battery) and using an acquisition frequency of 1 kHz. The behavior of the mice in the open field was video tracked for all recordings. On the baseline day, no kainate microinjection was performed, and the mice were recorded for 150 min. Over the next 5 days, the mice were recorded for 10 min before and 150 min after the kainate 0.75 ± 0.1 μL (100 μg/mL) (Sigma) microinjection [14]. For this procedure, mice were briefly anesthetized with 3–4% isoflurane, and the recording started 2–3 min thereafter.

### 2.4. Data Analysis

Dystonic motor behavior was evaluated, both on-site and online. A previously published scale was used to estimate the severity of symptoms [21,24] in which 0 = normal motor behavior; 1 = slightly abnormal motor behavior, no abnormal postures; 2 = minor motor impairment, abnormal postures when stimulated; 3 = moderate impairment, spontaneous dystonic postures that appear often; and 4 = severe disability, prolonged dystonic postures. The dystonia score was estimated for every 10 min. The total time of walking, active wake percentage (AW%), in the arena was expressed as a percentage of the recording’s whole time.

Data analysis included only recordings with no artifacts. ECoG data were visually evaluated to exclude contaminated recording intervals. Changes of coherence were evaluated on the baseline day, and before and after kainate for the next five consecutive days. The MATLAB function *mscohere* with 2 s windows overlapping 1 s from the 1 kHz signal was applied to obtain coherence values from power spectral density values of pairs from left to right primary motor cortex ECoGs. The results were then expressed by dividing them into frequency bands: delta: 0.5–3.5 Hz, theta: 4–12.5 Hz; beta: 13–30 Hz; low-gamma: 30.5–48 Hz; high-gamma: 52–100 Hz. Frequencies surrounding 50 Hz were not taken into consideration. The resulting data were processed in Microsoft Excel software, and then in GraphPad 8.4.3 for the performance of statistical tests. For all intervals, days and frequency bands, the D’Agostino and Pearson omnibus normality test was applied. The mixed-effects model (REML) test and Wilcoxon matched-pairs signed-rank test were used as the data was paired. For multiple comparisons, Dunn’s multiple comparison test was added to compare every kainate day to the baseline day.

Results are shown as mean ± the standard error of the mean (SEM), and a *p*-value < 0.05 was considered statistically significant. EMG analysis was comprised of calculating the power spectral density (from 1 to 200 Hz) with the Welch method with the *pwelch* MATLAB function. The imaginary part of coherence was calculated in order to exclude volume conduction [30]. Muscle activation parameters were calculated: mean power frequency and median frequency, root mean square and average rectified value. Furthermore, the relationship between behavior and interhemispheric coherence was evaluated by calculating the correlations between dystonia score, active wake percentage, and interhemispheric coherence per frequency band. The Pearson correlation coefficients (r) values and *p* values for significance were calculated. 

## 3. Results

### 3.1. Electromyography and Behavior Parameters Reveal Increased Muscular Activity after Cerebellar Kainate Injections

After the kainate cerebellar vermis application, mice showed impaired motor behavior. There was an increased dystonia score, decreased active wake percentages (Figure 1d), and increased muscle activation suggested by the EMG parameters, as we previously showed [15]. Mice usually remained in a prolonged muscle contraction for a few minutes, followed by periods of decreased symptoms. This phenotype resembles other studies using similar methods and is considered as dystonic [16]. EMG traces indicated increased activity during abnormal postures (Figure 1c).

### 3.2. Interhemispheric Coherence between Primary Motor Cortices before and after Repeated Cerebellar Vermis Kainate Application Was Reduced

**Figure 2:**
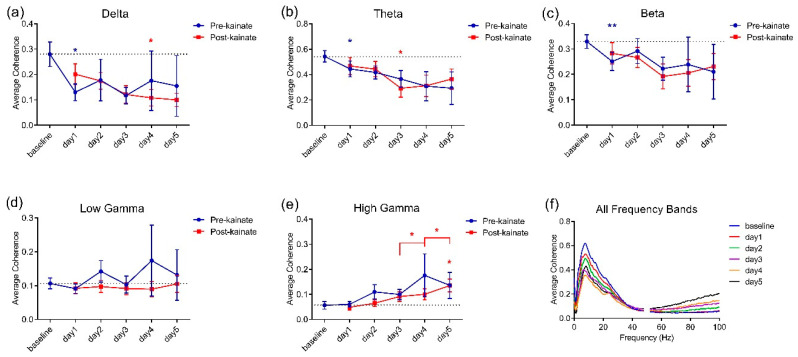
Average interhemispheric coherence between primary motor cortices before (baseline day) and after 5 days of repeated vermis cerebellar kainate administration across different frequency spectral bands. (**a**) Delta band (0.5–3.5 Hz). (**b**) Theta band (4–12.5 Hz). (**c**) Beta band (13–30 Hz). (**d**) Low-gamma band (30.5–48 Hz). (**e**) High-gamma band (52–100 Hz) frequencies in baseline day and kainate administration day. (**f**) For each frequency band, the coherences are expressed as average ± the standard error of the mean (* for *p* < 0.05 and ** for *p* < 0.01, day of kainate application versus baseline day; statistical tests detailed in Table 1).

**Figure 3:**
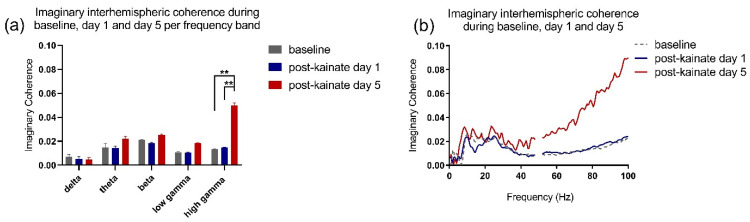
(**a**) Imaginary interhemispheric coherence during baseline and kainate day per frequency band. Results are expressed as average ± the standard error of the mean. (**b**) Imaginary interhemispheric coherences across 0.5–100 Hz frequencies (average) (** for *p* < 0.01).

When comparing baseline day values to the values from the days of kainate application on the cerebellar vermis, the left–right motor cortices coherence was reduced in low-frequency bands (Figure 2a–c). The differences were significant in delta and theta bands in kainate days 4 and 3, respectively (Figure 2a,b). In the high gamma band, the coherence decreased slightly on the first day of kainate application and then gradually increased until day 5, when the change was significant (Figure 2e). We also observed that after day 3, there was a rising trend in theta, beta, and low gamma bands in days 4 and 5, with a significant change only for the high gamma band. The recordings in the pre-kainate state had a similar trend, with a decrease in the delta, theta, and beta bands, with significant changes for day 1, and a tendency to increase in the high-gamma band, suggesting a possible compensatory mechanism. Statistical indicators are shown in Table 1. The imaginary part of coherence did not show volume conduction (Figure 3a,b), according to the statistical indicators presented in Table 2. 

### 3.3. Correlations between Motor Behavior and Interhemispheric Coherence between Primary Motor Cortices

**Figure 4:**
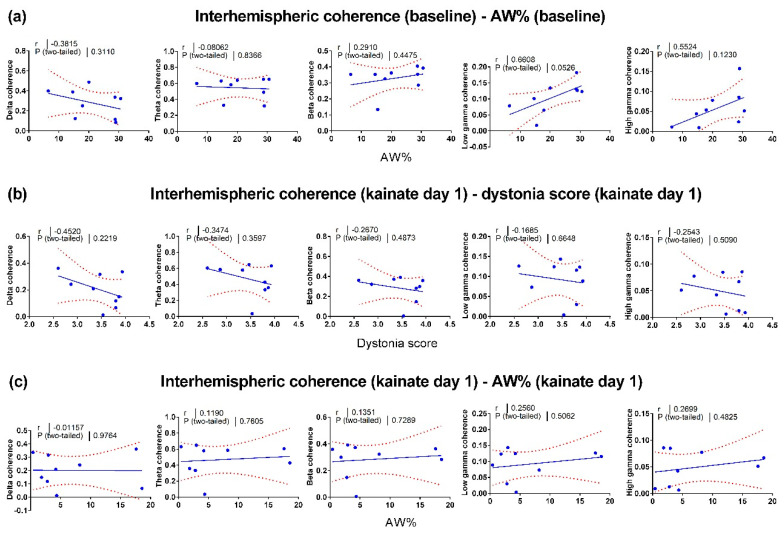
Pearson correlations between left and right motor cortices interhemispheric coherence and behavior in baseline and the first day of cerebellar kainate application. (**a**) Linear regression of active wake percentage in baseline day versus interhemispheric coherence (delta, theta, beta, low gamma, high gamma bands). (**b**) Linear regression of dystonia score versus interhemispheric coherence (delta, theta, beta, low gamma, high gamma bands) in kainate day 1. (**c**) Linear regression of active wake percentage versus interhemispheric coherence (delta, theta, beta, low gamma, high gamma bands) in kainate day 1. Each point corresponds to one subject. The plain and dotted curves denote the 95% tolerance and confidence intervals of the linear regression, correspondingly. R and *p* values are indicated above on each graph.

**Figure 5:**
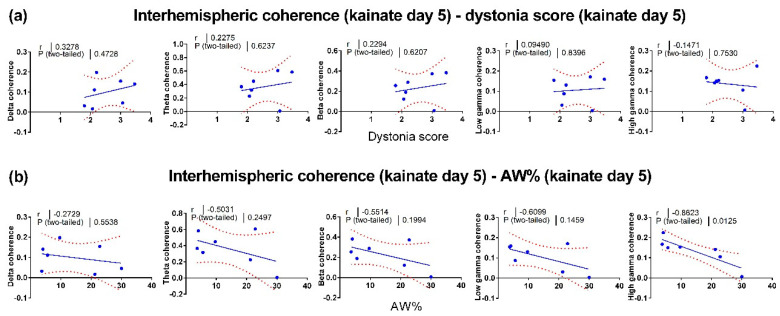
Pearson correlations between left and right motor cortices (interhemispheric) coherence and behavior on the fifth day of cerebellar kainate application. (**a**) Linear regression of dystonia score in kainate day 5 versus interhemispheric coherence (delta, theta, beta, low gamma, high gamma bands). (**b**) Linear regression of active wake percentage versus interhemispheric coherence (delta, theta, beta, low gamma, high gamma bands) in kainate day 5. Each point corresponds to one subject. The plain and dotted curves denote the 95% tolerance and confidence intervals of the linear regression, correspondingly. R and *p* values are indicated above on each graph.

To find a possible connection between the neural activity changes and motor behavior changes, we evaluated the relationship between locomotor activity and interhemispheric coherence. Firstly, we assessed the baseline correlations (Figure 4a). Then, we evaluated the correlations between the dystonia score (Figure 4b), the active wake percentage (Figure 4c) versus the interhemispheric coherence from the first kainate administration day. Additionally, we calculated the correlations between the dystonia score (Figure 5a) and active wake percentages (Figure 5b) versus interhemispheric coherence per frequency band on day 5. We found little or no relationship between behavior and cortical interhemispheric communication for both kainate day 1 and kainate day 5. One possible explanation is the lack of variability of dystonia scores and the small number of animals. Average dystonia scores were high due to the severity of the phenotype after the kainate administration (Figure 1d).

## 4. Discussion

The cerebellum is implicated in motor control and cognitive functions as it receives input through the cerebro-ponto-cerebellar pathway and the cerebro-olivo-cerebellar pathway [31] and sends projections to the prefrontal, premotor and primary motor cortex [32]. These anatomical connections are functionally meaningful, and the cerebellum is suggested to be a gateway that modulates motor circuits and cortical oscillatory activity [24].

The cortical electrical activity varies over the cortex and in a specific area when performing a motor task. Quantitative ECoG gives us insights into the communication between brain regions. Power spectral analysis of the ECoG signal assesses the primary cerebral rhythms and reflects the cortical activation or inhibition. Furthermore, coherence is a useful parameter that measures the synchronicity between two oscillatory signals. Coherence is also used as a marker of the functional coupling of different brain regions [33,34,35]. Increased coherence between two ECoG signals suggests a synchronized neuronal activity, thus increased communication between the recorded brain areas. Low coherence suggests neuronal populations that are acting independently, so they have decreased functional connectivity in between them.

In this study, we examined the influence of cerebellar vermis activation with kainate applied on the dura mater over the interhemispheric communication between motor cortices, assessed as motor cortices ECoG coherence. We found that kainate triggers behavioral and motor symptoms characteristic to dystonia quantifiable by the dystonia score, active wake % and EMG parameters [24]. A decrease in the left–right motor cortex coherence was found across all spectral frequency bands, but significant changes were found only for delta and theta bands. These modifications suggest that the cerebellum has a strong influence over these brain areas, revealing a capacity to modulate motor cortex oscillatory activity. No significant correlations were found between behavior and coherence between left and right primary motor cortices per band of frequency. Furthermore, after the repeated triggering of the dystonic attacks, we found a functional adaptation of the interhemispheric coherence throughout the days of the kainate application. Likewise, processes of compensation have been found to take place for the motor-parietal and motor-sensorimotor coherences after repeated cerebellar vermis kainate application [24]. Thus, compensatory activity and motor circuit plasticity occur when the output of the cerebellum is impaired.

The ECoG, EMG, and behavioral changes induced by kainate microinjection on the cerebellar vermis are mediated by the cerebellar Purkinje cells as the mice lacking Purkinje cells did not display motor behavior abnormalities [21]. The Purkinje cells in the cerebellar cortex process the input signals from the cortico-cerebellar pathways through the climbing fibers and mossy fibers and integrate the local inhibitory signals of the stellate and basket cells, to modulate the cerebellar nuclei neurons activity through GABA-ergic synapses [36]. Therefore, Purkinje cell stimulation results in a reduced cerebellar excitatory output to the motor cortex. The cerebello-thalamo-cortical pathway terminates on both inhibitory and excitatory neurons in the motor cortex. Thus, the reduced cerebellar output may increase or decrease the inhibition within the motor cortex [37].

Movements vary from simple to complex motor tasks, during mice exploratory activity or grooming behavior. They involve the activation of an entire network of cortical and subcortical areas: cerebellum, thalamus, basal ganglia, pons, and sensory-motor cortices. All of these centers should coordinate their activity within the network and with other neural networks to have a normal, precise, and correct movement. The left–right motor cortices coherence during separate movements of the wrists showed a higher coherence when moving the right wrist, for all right-handed subjects. In addition, the power suppression was higher in the left motor cortex during the right wrist movements than the power suppression done by the right motor cortex during left wrist movements.

Moreover, a strong modulation for cerebro-muscular coherence was observed in the gamma band (26–40 Hz), especially in isometric contraction conditions. However, strong variations were noticed in the beta band (13–24 Hz) between static and dynamic conditions, suggesting a functional role of oscillatory activity coupling in the motor system [38]. Low-frequency oscillations participate in sensory-motor control and the formation of memories, also occurring during sleep. They are thought to spread through cerebro-cerebellar loops comprising multiple structures, such as the prefrontal cortex, sensory-motor cortex, premotor area, posterior parietal cortex, thalamus, and the cerebellum, ultimately returning to the cerebral cortex. The integrity of this loop ensures the generation of the movement sequence, which requires attention, decision making, and sensory-motor control. Voluntary movements can trigger oscillatory cortical activity in the prefrontal regions that propagate to all structures, using the cerebellum as a relay [3].

Interestingly, during bimanual force generation, when the isometric contraction was voluntary, the motor cortices coherence decreased in the 8–13 Hz band. The changes in oscillatory activity in this frequency band, in humans, called the alpha band, are interpreted as being involved in the suppression of unwanted EMG activity, correction of movement error, or suppression of task-irrelevant activity during finger movements [39]. Similarly, in our study, there was a significant decrease in theta band coherence, which includes the 8–13 Hz frequency interval. During the dystonic behavior, there were mainly tonic contractions of the muscles on both sides of the body.

Furthermore, dysfunctions of the cortico-subcortical circuits have been associated with multiple psychiatric phenotypes. The basal ganglia-thalamo-cortical circuits have been shown to contribute to the development of multiple behavioral and cognitive impairments. One explanation might be that motor abnormalities can influence the fronto-striatal circuitry, whose dysfunction can trigger affective disorders by altering the limbic circuitry [40]. Primary focal dystonia is a genetic disorder in which decreased amounts of dopamine can be found in the nigrostriatal neurons [41], representing a consequence of an anomalous cortico-striatal circuitry. Recent evidence has shown that social phobia, social anxiety, and obsessive-compulsive disorder are significantly higher in this pathology [42]. Patients with primary focal dystonia show distinct neuropsychiatric and personality profiles on the anxiety spectrum, such as obsessive-compulsive and avoidant personality disorders, and often manifest social phobia, agoraphobia, and panic disorder [43].

A faulty interhemispheric communication was also noticed in several other neurobehavioral disorders, such as ADHD, autism (including Asperger’s syndrome), and Tourette’s syndrome. Strikingly, individuals affected by these types of disorders show skills at different levels of development and performance, indicating a functional disconnection syndrome, where enhanced abilities occur with increased network coherence and reduced abilities with low network coherence [2]. This dissymmetry can sometimes be associated with anatomical asymmetries [44], or brain regions with tardy development. Interestingly, there is evidence supporting the fact that individuals diagnosed with autism, ADHD, and Asperger’s syndrome present with asymmetrical brain regions and reduced coherences between corresponding left–right structures. Another change is the activation of the right hemisphere, which is responsible for cognitive, motor, sensory, and autonomic functions. The impairments found in autistic spectrum disorder may come from the preferential use of the local networks and the impossibility of synchronizing the activity of the two hemispheres, more so as the left hemisphere is mainly specialized in local processing and the right hemisphere in global processing. People affected by the mentioned disorders present with decreased motor and cognitive abilities, especially regarding language and thought, such as motor planning, working memory, abstract reasoning, cognitive flexibility, temporal sequencing, and generativity. Dopamine is considered the principal neurotransmitter involved in the development of executive intelligence and the control of the skills associated with the left hemisphere [45]. Thus, it was proposed that a unilateral (dopaminergic) increased hemispheric stimulation can induce temporal oscillations within the thalamo-cortical pathway, creating a compensatory oscillatory cortical activity, similar to the one found in the functional hemisphere. Increasing the baseline oscillation of a hemisphere can improve the coordination and coherence between the hemispheres, and thus enhance motor and cognitive abilities [2].

The highly increased dopaminergic activity was also observed in the mesolimbic areas of obsessive-compulsive [46] and schizophrenic [47] patients. In contrast, in Tourette’s syndrome, excessive dopamine was found in the basal ganglia, as well as the thalamus, striatum and fronto-cortical circuits [48]. Moreover, mania was found in persons who exhibited excessive dopamine in both medial and lateral dopaminergic systems [49]. However, the thalamo-cortical circuits seem to be highly implicated in maintaining balance and coordination across different areas in the brain, a function exerted by keeping a temporal coherence between implicated regions, mostly sensory, motor and association areas, even though they are located very far from each other. All of the above mentioned regions provide a feedforward flow of information. In humans, there is evidence showing that 40–50 Hz or even higher oscillatory rates, are the rates at which attention-demanding cognitive tasks are best processed [50]. This gamma-band local synchronization can facilitate neural communication in several regions implicated in language, vision, and memory, but also in sensorimotor processes [51]. The neuronal activity found in association areas, including prefrontal, posterior parietal, inferotemporal cortex, and other subcortical regions, show that the top-down influences can shape the intrinsic dynamic of thalamo-cortical networks. This activity creates a predictive nature of self-generated fluctuations of neuronal excitability levels in these areas, proving that maintaining a proper coherence among the related structures is especially important [52]. Before movements are executed, motor and premotor areas activate [53], helping the selection of response, setting the movement parameters (direction, latency, speed), and coordinating specific neural populations that are needed for the dynamic organization of that movement [54]. These processes are usually related to the same modulation source, often the prefrontal cortex [55]. Besides the firing rate modulation that occurs during this phenomenon, changes in neuronal synchrony also happen, especially for the motor and premotor neuronal networks [56].

It is known that a more active hemisphere or one operating at a different frequency of oscillation can alter the hemispheres’ ability to synchronize and maintain a temporal coherence between large areas of the brain, thus hindering the connectivity between distant neurons [2]. Moreover, in humans, positron-emission tomography studies show an additional essential role of the cerebellum in cognitive functions, being part of the cerebello-thalamic-prefrontal network supporting various mental activities that require gamma-band synchrony and temporal, fluid coordination of thoughts. This is also indicated by the fact that this network becomes dysfunctional in several cognitive-affective disorders, such as schizophrenia [57].

## 5. Conclusions

The results of the present study show low coherences between the left and right motor cortices for delta, theta and beta bands, and abnormal postures and repetitive movements when perturbing the cerebellar output. Therefore, the cerebello-thalamo-prefrontal circuits might play a considerable role in maintaining synchrony between the two hemispheres. Our study offers novel insights about the cerebellum’s contribution to interhemispheric communication and how cerebellar activation modulates the motor cortices connectivity during dystonic postures and movements.

## Figures and Tables

**Table 1:** Interhemispheric coherence between primary motor cortices. Statistical data (Figure 2a–f).

Interhemispheric Coherence between Primary Motor Cortices		
Frequency Band	Test	*p*-Value
**Delta pre-kainate**	Mixed-effects model (REML) F (1.953, 15.23) = 0.6940	0.5114
day 0 vs. day 1	Dunnett’s multiple comparisons test (^1^)	0.0125 (*)
day 0 vs. day 2	^1^	0.8189
day 0 vs. day 3	^1^	0.0874
day 0 vs. day 4	^1^	0.8051
day 0 vs. day 5	^1^	0.6919
**Delta post-kainate**	Mixed-effects model (REML) F (1.662, 12.30) = 4.097	0.0492 (*)
day 0 vs. day 1	^1^	0.0752
day 0 vs. day 2	^1^	0.3924
day 0 vs. day 3	^1^	0.1531
day 0 vs. day 4	^1^	0.0499 (*)
day 0 vs. day 5	^1^	0.0800
**Theta pre-kainate**	Mixed-effects model (REML) F (1.553, 12.11) = 1.768	0.2129
day 0 vs. day 1	^1^	0.0135 (*)
day 0 vs. day 2	^1^	0.0807
day 0 vs. day 3	^1^	0.0833
day 0 vs. day 4	^1^	0.1715
day 0 vs. day 5	^1^	0.1715
**Theta post-kainate**	Mixed-effects model (REML) F (2.134, 15.37) = 5.300	0.0164
day 0 vs. day 1	^1^	0.0985
day 0 vs. day 2	^1^	0.1417
day 0 vs. day 3	^1^	0.0262
day 0 vs. day 4	^1^	0.0868
day 0 vs. day 5	^1^	0.1221
**Beta pre-kainate**	Mixed-effects model (REML) F (1.538, 12.00) = 0.5601	0.5414
day 0 vs. day 1	^1^	0.0029 (**)
day 0 vs. day 2	^1^	0.9615
day 0 vs. day 3	^1^	0.0986
day 0 vs. day 4	^1^	0.8681
day 0 vs. day 5	^1^	0.7087
**Beta post-kainate**	Mixed-effects model (REML) F (2.265, 16.76) = 2.576	0.1006
day 0 vs. day 1	^1^	0.1348
day 0 vs. day 2	^1^	0.1190
day 0 vs. day 3	^1^	0.1300
day 0 vs. day 4	^1^	0.1999
day 0 vs. day 5	^1^	0.1267
**Low gamma pre-kainate**	Mixed-effects model (REML) F (0.9834, 7.670) = 0.7102	0.4225
day 0 vs. day 1	^1^	0.2172
day 0 vs. day 2	^1^	0.7971
day 0 vs. day 3	^1^	0.9998
day 0 vs. day 4	^1^	0.9465
day 0 vs. day 5	^1^	0.9955
**Low gamma post-kainate**	Mixed-effects model (REML) F (1.458, 10.79) = 0.6411	0.4982
day 0 vs. day 1	^1^	0.5557
day 0 vs. day 2	^1^	0.7443
day 0 vs. day 3	^1^	0.7968
day 0 vs. day 4	^1^	0.6618
day 0 vs. day 5	^1^	0.9999
**High gamma pre-kainate**	Mixed-effects model (REML) F (1.253, 9.772) = 1.800	0.2140
day 0 vs. day 1	^1^	0.9370
day 0 vs. day 2	^1^	0.4573
day 0 vs. day 3	^1^	0.1390
day 0 vs. day 4	^1^	0.5823
day 0 vs. day 5	^1^	0.4789
**High gamma post-kainate**	Mixed-effects model (REML) F (2.549, 18.86) = 5.603	0.0084 (**)
day 0 vs. day 1	^1^	0.8771
day 0 vs. day 2	^1^	0.9507
day 0 vs. day 3	^1^	0.5170
day 0 vs. day 4	^1^	0.1885
day 0 vs. day 5	^1^	0.0102 (*)
**Delta pre-kainate**		
day 3 vs. day 4	Wilcoxon matched-pairs signed rank test (^2^)	0.6406
day 4 vs. day 5	^2^	0.2969
**Delta post-kainate**		
day 3 vs. day 4	^2^	0.8438
day 4 vs. day 5	^2^	0.2188
**Theta pre-kainate**		
day 3 vs. day 4	^2^	0.3828
day 4 vs. day 5	^2^	0.5781
**Theta post-kainate**		
day 3 vs. day 4	^2^	>0.9999
day 4 vs. day 5	^2^	0.4375
**Beta pre-kainate**		
day 3 vs. day 4	^2^	0.7422
day 4 vs. day 5	^2^	0.1094
**Beta post-kainate**		
day 3 vs. day 4	^2^	>0.9999
day 4 vs. day 5	^2^	0.3750
**Low gamma pre-kainate**		
day 3 vs. day 4	^2^	0.8438
day 4 vs. day 5	^2^	0.4688
**Low gamma post-kainate**		
day 3 vs. day 4	^2^	>0.9999
day 4 vs. day 5	^2^	0.3750
**High gamma pre-kainate**		
day 3 vs. day 4	^2^	0.1953
day 4 vs. day 5	^2^	>0.9999
**High gamma post-kainate**		
day 3 vs. day 4	^2^	0.0156(*)
day 4 vs. day 5	^2^	0.0313(*)

Legend: * *p* < 0.05, ** *p* < 0.01.

**Table 2:** Imaginary interhemispheric coherence between primary motor cortices. Statistical data (Figure 3a,b).

Imaginary Interhemispheric Coherence between Primary Motor Cortices		
Frequency Band	Test	*p*-Value
**Delta post-kainate**	Mixed-effects model (REML) F (0.9522, 7.617) = 0.3628	0.5541
day 0 vs. day 1	Tukey’s multiple comparisons test (^3^)	0.8356
day 0 vs. day 5	^3^	0.9514
day 1 vs. day 5	^3^	0.9885
**Theta post-kainate**	Mixed-effects model (REML) F (0.8259, 6.607) = 0.001127	0.9543
day 0 vs. day 1	^3^	0.9019
day 0 vs. day 5	^3^	0.9617
day 1 vs. day 5	^3^	0.8733
**Beta post-kainate**	Mixed-effects model (REML) F (1.316, 10.53) = 0.2092	0.7226
day 0 vs. day 1	^3^	0.5070
day 0 vs. day 5	^3^	0.9933
day 1 vs. day 5	^3^	0.7590
**Low gamma post-kainate**	Mixed-effects model (REML) F (0.9655, 7.724) = 2.428	0.1591
day 0 vs. day 1	^3^	0.6644
day 0 vs. day 5	^3^	0.5433
day 1 vs. day 5	^3^	0.0896
**High gamma post-kainate**	Mixed-effects model (REML) F (1.082, 8.658) = 17.85	0.0022 (**)
day 0 vs. day 1	^3^	0.7502
day 0 vs. day 5	^3^	0.0093 (**)
day 1 vs. day 5	^3^	0.0028 (**)

Legend: ** *p* < 0.01.
